# Chemical characterization of inks in skin reactions to tattoo

**DOI:** 10.1107/S1600577522008165

**Published:** 2022-10-04

**Authors:** Hester Colboc, Dominique Bazin, Solenn Reguer, Ivan T. Lucas, Philippe Moguelet, Reyhan Amode, Chantal Jouanneau, Angèle Soria, François Chasset, Emmanuelle Amsler, Catherine Pecquet, Sélim Aractingi, Ludovic Bellot-Gurlet, Lydia Deschamps, Vincent Descamps, Nicolas Kluger

**Affiliations:** a Sorbonne Université, Hôpital Rothschild, Service de Gériatrie-Plaies et Cicatrisation, Paris, France; b Sorbonne Université, UPMC Paris 06, Institut National de la Santé et de la Recherche Médicale, Unité Mixte de Recherche S 1155, F-75020 Paris, France; c Institut de Chimie Physique, Université Paris-Saclay, Orsay, France; d DiffAbs Beamline, Synchrotron SOLEIL, L’Orme des Merisiers, Départementale 128, 91190 Saint-Aubin, France; e Sorbonne Université, CNRS, Laboratoire Lise UMR 8235, Paris, France; f Sorbonne Université, Hôpital Tenon, Anatomie et Cytologie Pathologiques, Paris, France; g Private Practice, Paris, France; h Sorbonne Université, Hôpital Tenon, Service de Dermatologie-Allergologie, Paris, France; i Université de Paris, Hôpital Cochin, Service de Dermatologie, Paris, France; j Sorbonne Université, UMR 8233, Paris, France; k Université de Paris, Hôpital Bichat, Service d’Anatomie et Cytologie Pathologique, Paris, France; l Université de Paris, Hôpital Bichat, Service de Dermatologie, Paris, France; m Department of Dermatology, Allergology and Venereology, Helsinki University Hospital and University of Helsinki, Helsinki, Finland; University of Essex, United Kingdom

**Keywords:** tattoos, cutaneous hypersensitivity reactions, X-ray fluorescence spectroscopy, X-ray absorption spectroscopy, Raman spectroscopy

## Abstract

Skin reactions are well described complications of tattooing, usually provoked by red inks. Chemical characterizations of these inks are usually based on limited subjects and techniques. This study aimed to determine the organic and inorganic composition of inks using X-ray fluorescence spectroscopy (XRF), X-ray absorption spectroscopy (XANES) and Raman spectroscopy, in a cohort of patients with cutaneous hypersensitivity reactions to tattoo. A retrospective multicenter study was performed, including 15 patients diagnosed with skin reactions to tattoos. Almost half of these patients developed skin reactions on black inks. XRF identified known allergenic metals – titanium, chromium, manganese, nickel and copper – in almost all cases. XANES spectroscopy distinguished zinc and iron present in ink from these elements in endogenous biomolecules. Raman spectroscopy showed the presence of both reported (azo pigments, quinacridone) and unreported (carbon black, phtalocyanine) putative organic sensitizer compounds, and also defined the phase in which Ti was engaged. To the best of the authors’ knowledge, this paper reports the largest cohort of skin hypersensitivity reactions analyzed by multiple complementary techniques. With almost half the patients presenting skin reaction on black tattoo, the study suggests that black modern inks should also be considered to provoke skin reactions, probably because of the common association of carbon black with potential allergenic metals within these inks. Analysis of more skin reactions to tattoos is needed to identify the relevant chemical compounds and help render tattoo ink composition safer.

## Abstract 2

Using X-ray fluorescence spectroscopy, X-ray absorption spectroscopy and Raman spectroscopy, the chemical composition of inks was analyzed in a cohort of patients with cutaneous hypersensitivity reactions to tattoo. Known inorganic allergenic metals (titanium, chromium, manganese, nickel and copper) were identified in almost all cases as well as azo pigments, quinacridone, carbon black and phthalocyanine.

## 1. Introduction

Tattooing refers to introducing pigmented material into the dermis by puncturing the skin, to obtain a permanent symbolic or decorative design (Wohlrab *et al.*, 2007 ▸). This practice dates back thousands of years, and probably started around the Neolithic period (Oeggl *et al.*, 2007 ▸). In Europe, the number of tattooed people is estimated to be about 100 million (Kluger *et al.*, 2019 ▸). Although the tattoo fashion has thus been around for centuries, the question of its health impact has really arisen lately (Bäumler, 2016 ▸). In fact, tattoos are associated with various complications, with allergic reactions dominating (Islam *et al.*, 2016 ▸; Serup *et al.*, 2015 ▸), which can strongly influence quality of life (Hutton Carlsen & Serup, 2015 ▸).

Tattoo inks are composed of pigments combined with additives such as dispersants and preservatives (Liszewski & Warshaw, 2019 ▸; Timko *et al.*, 2001 ▸). Once the tattoo is performed, skin tissue is permanently exposed to these substances for life. Nowadays, natural pigments are very rarely used and most tattoo inks are synthetic, allowing cheaper mass production and a large variety of shades.

Tattoo ink composition is poorly regulated worldwide. In Europe, only a few recent guidelines exist. In 2008, the European Council passed a resolution regarding the composition of tattoo inks and permanent make-up (Piccinini *et al.*, 2008 ▸). However, Hauri *et al.* reported that up to one third of tattoo inks contain prohibited pigments, illustrating the fact that these recommendations are unfortunately poorly observed (Hauri & Hohl, 2015 ▸; Desmedt *et al.*, 2022 ▸).

Compounds in tattoo inks may be classified into two types: inorganic substances, such as iron, zinc and titanium and their oxides (Tighe *et al.*, 2017 ▸), and organic substances, including polycyclic aromatic hydro­carbons (PAHs) mainly found in black inks (Regensburger *et al.*, 2010 ▸), organic azo-colorants and quinacridone found in colored inks (Laux *et al.*, 2016 ▸). Once in the skin, the original pigments can undergo metabolic changes, leading to the formation of other compounds, such as primary aromatic amines (PAA), cleavage products of azo-colorants (Laux *et al.*, 2016 ▸). These metabolic changes are suspected to be the consequences of various phenomena, including enzymatic activity, thermal decomposition, or photodegradation occurring after UV light exposure or laser tattoo removal (Cui *et al.*, 2005 ▸; Engel *et al.*, 2007 ▸; Vasold *et al.*, 2004 ▸). Some of these inorganic and organic compounds, as well as cleavage products of the latter, are known to be allergenic, carcinogenic, mutagenic and reproduction-toxic substances (Laux *et al.*, 2016 ▸; Manso *et al.*, 2019 ▸). Studies analyzing the relationship between color and allergic reactions show that colored tattoos, especially red, cause more allergic reactions than black (Serup *et al.*, 2020 ▸; Kluger, 2016 ▸).

Previous skin biopsy studies of *in situ* tattoo ink reactions identified multiple compounds: quinacridones (Serup *et al.*, 2020 ▸; Gaudron *et al.*, 2015 ▸), azo-pigments (Serup *et al.*, 2020 ▸; Gaudron *et al.*, 2015 ▸; Schreiver *et al.*, 2017 ▸; Hutton Carlsen *et al.*, 2016 ▸), phthalocyanine (Serup *et al.*, 2020 ▸) and metal oxides, usually used as shading agents (Serup *et al.*, 2020 ▸; Schreiver *et al.*, 2017 ▸, 2019 ▸). Using sector field inductively coupled plasma mass spectrometry, Forte *et al.* (2009*a* ▸) identified and quantified Cr (highest concentration, 315–4720 ng g^−1^), Ni (37.5–2318 ng g^−1^) and Cd (6.67–1150 ng g^−1^) in 13 tattoo inks, with Cr and Ni being well known allergenic metals (Thyssen *et al.*, 2011 ▸). Involvement of these metals in skin reactions to tattoos has also been shown *in vivo*: De Cuyper *et al.* (2017 ▸), for example, have reported a patient with papulo-nodular infiltration of his tattoo that disappeared within a week after surgical removal of metal osteosynthesis implants. Nickel, chromium and titanium were identified both within the skin biopsy and the implant. However, these studies were either limited to a few techniques, or included a limited number of patients.

Wide-ranging chemical characterizations have been performed directly on tattoo inks (Arl *et al.*, 2019 ▸; Poon *et al.*, 2008 ▸; Forte *et al.*, 2009*a* ▸), pigments (Yakes *et al.*, 2016 ▸) or skin samples from tattooed animals (Poon *et al.*, 2008 ▸). Unfortunately, these studies are not directly relevant to human skin reactions; because of the specific metabolic changes tattoo inks undergo once introduced into human skin, it is clearly more pertinent to perform analyses on human skin samples themselves.

The present study aims to determine both the organic and inorganic chemical composition of inks in skin samples using multi-scale and multi-technique approaches on a cohort of patients with cutaneous reactions to tattoos. A specific approach has already been developed in related works and applied to different kinds of abnormal deposits identified in different parts of the human body (Bazin *et al.*, 2012 ▸, 2016 ▸). We have also applied this methodology to skin (Colboc *et al.*, 2016 ▸, 2019*a* ▸,*b* ▸, 2020 ▸, 2022*a* ▸,*b* ▸) as described below. Elemental composition and distribution of chemical elements can be determined using X-ray fluorescence (XRF) (Rouzière *et al.*, 2016 ▸; Reguer *et al.*, 2016 ▸), while chemical analyses are performed using a combination of Raman (Lucas *et al.*, 2022 ▸) and X-ray absorption spectroscopy (mainly XANES, X-ray absorption near-edge structure) (Bazin *et al.*, 2022*a* ▸).

## 2. Materials and methods

### 2.1. Patients

This study complied with Good Clinical Practices and the Declaration of Helsinki, and French law. We conducted a retrospective multicenter study in Tenon and Bichat Hospitals (Paris, France) and the Skin and Allergies Hospital (Helsinki, Finland) from January 2001 to December 2017. Patients diagnosed with cutaneous reactions to tattoo were included and their clinical and histopathological data collected.

### 2.2. Skin biopsies preparation

Skin biopsy samples from these patients were all centralized in the Department of Pathology of Tenon Hospital. For each sample, 1.5 µm-thick sections from paraffin-embedded skin biopsies were deposited on both glass slides for hematoxylin–eosin–saffron (HES) staining and low-e microscope slides (MirrIR, Kevley Technologies, Tienta Sciences, Indianapolis, IN, USA) for Raman spectroscopy (Dessombz *et al.*, 2011 ▸; Bazin & Daudon, 2015 ▸). XRF and XANES measurements were performed directly on the whole thickness of the paraffin-embedded tissue blocks. The entire block was analyzed except for three larger samples where a selected part of the sample was studied.

### 2.3. Characterization techniques

#### 2.3.1. X-ray synchrotron based techniques

All X-ray based synchrotron experiments were performed at room temperature and atmospheric pressure on the DiffAbs beamline at the SOLEIL synchrotron facility (France), which offers multi-technique and multi-scale characterization of various materials (Reguer *et al.*, 2016 ▸). This beamline provides a monochromatic X-ray beam, tunable in the 3–23 keV energy range. The Si(111) double-crystal monochromator is positioned between two cylindrical vertically focusing mirrors in order to monochromatize and focus the beam to about 300 µm diameter. A secondary optical system consisting of two trapezoidal orthogonally positioned curved mirrors under grazing incidence allows the X-ray beam to be focused down to a 5–10 µm-diameter beam spot with a flux close to 10^10^ photons s^−1^ at the sample position. In the present study, all samples were analyzed using a beam spot of 8 µm × 4 µm (horizontal × vertical, full width at half-maximum). XRF and XANES combined measurements were applied to highlight the localization and distribution of the chemical elements as well as the local environment of probed atoms (Dessombz *et al.*, 2013 ▸; Bazin *et al.*, 2022*a* ▸,*b* ▸).

Points of interest were selected on XRF maps, to measure Zn and Fe *K*-edge XANES spectra. The energy range for the spectral acquisition at the Zn *K*-edge was selected between 9.63 and 10 keV, with an energy step of 1 eV in the pre-edge and post-edge domain but 0.5 eV near the edge and a 1 s acquisition time. This *K*-edge results in a Zn signal more prominent than that of other elements, such as Ca or Ti, but does not indicate higher Zn content. Various correction procedures have to account for the self-absorbing matrix and the fact that measurements have been performed in air, the nature of the matrix, absorption by air, incident beam energy, and the ionization and X-ray emission cross sections associated with each element (Bazin *et al.*, 2021 ▸).

The energy range for the spectral acquisition at the Fe *K*-edge was selected between 7.050 and 7.255 keV with an energy step of 1 eV in the pre-edge and post-edge region but 0.5 eV close to the edge and a 1 s acquisition time. XRF and XANES spectra were acquired using a four-element silicon drift detector (Vortex-ME4, Hitachi). Particular attention was paid to the data analysis which takes into account the shape and position of the edge, and the presence of pre-edge and post-edge first oscillation. These features indicate the effective charge of the atoms as well as the spatial distribution of atoms around Fe and Zn (Bazin *et al.*, 2022*a* ▸).

#### 2.3.2. Raman spectroscopy

Raman spectroscopy was performed in order to characterize the organic as well as inorganic substances present within the tattoo inks (Yakes *et al.*, 2016 ▸). It also allowed us to determine the chemical phase in which some of the metals are engaged, such as Ti cations which can be present as anatase or rutile (Frank *et al.*, 2012 ▸). Spectra were collected using a micro-Raman system (LabRam HR-800 Evolution, Horiba, Japan) with a 785 nm laser excitation wavelength, 100× objective (Olympus, numerical aperture 0.9) and 300 grooves per mm grating (Lucas *et al.*, 2022 ▸; Tamosaityte *et al.*, 2021 ▸; Carden *et al.*, 2003 ▸).

Raman spectra of skin biopsy may display strong auto-fluorescence (as do many biological samples with protein matrices). In such cases, Raman signatures can be dominated or even totally masked by the fluorescence background, making their detection and analysis impossible. This experimental complication dictates a need to pay special attention to the wavelength of the incident laser.

## 3. Results and discussion

### 3.1. Relationship between cutaneous reactions, ink color and histopathological data

**Figure 1:**
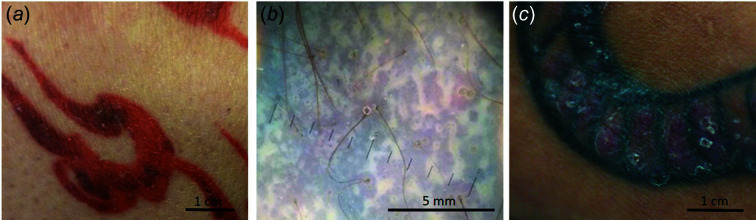
Clinical presentations of skin reactions on tattoo. (*a*) Patient 1, presenting a papular eruption on a red tattoo. (*b*) Patient 9, presenting a lichenoid eruption on a black tattoo (picture taken using a dermatoscope). (*c*) Patient 10, presenting a papular eruption on a black tattoo.

Fifteen patients, mean age 32 [20–62], 9 men and 6 women, were included. All had developed papular erythematous eruption [Figs. 1 ▸(*a*), 1 ▸(*c*) and 1 ▸(*d*)] or lichenoid eruption [Fig. 1 ▸(*b*)] associated with itching and swelling 0.5 to 60 months following the tattoo procedure. Clinical and histopathological data are summarized in Table 1 ▸. Red ink was involved in 7 cases, black in 7, and blue in 1. These reactions were confined to one specific color when the tattoo contained multiple colors [Fig. 1 ▸(*c*)], suggesting the presence of a specific sensitizer compound in one of the inks used. Optical microscopy always identified the presence of intra-dermal colored pigments, associated with the histological reaction pattern which presented as lichenoid (6), lympho-histiocytic (5) or granulomatous (4).

In a critical review, Thum *et al.* showed that inflammatory complications related to tattooing are associated with a wide spectrum of histological reaction patterns. Consistent with what they described, and probably because of the variety of chemical substances present in modern tattoo inks, we could not directly associate a type of histopathological reaction to a specific color (Thum & Biswas, 2015 ▸).

### 3.2. Chemical information from XRF

One of the major strengths of µXRF is its ability to simultaneously determine and locate a large number of major and trace elements in a biopsy (Estève *et al.*, 2016*a* ▸,*b* ▸, 2017 ▸). Furthermore, because we analyzed the full thickness of the paraffin-embedded skin biopsies, we were able to gather information on the entire sample (in contrast to Raman analysis performed on sections only 1.5 µm thick).

Various parameters allowed a correlation between optical microscopy results and XRF spectra. Calcium was used as a marker of the epidermis within the skin biopsy, as one of its layers, the stratum granulosum, is known to have a high concentration of calcium (Lee & Lee, 2018 ▸). Correlation was also made between skin vessels observed in HES staining and the linear Fe signal in XRF maps.

**Figure 2:**
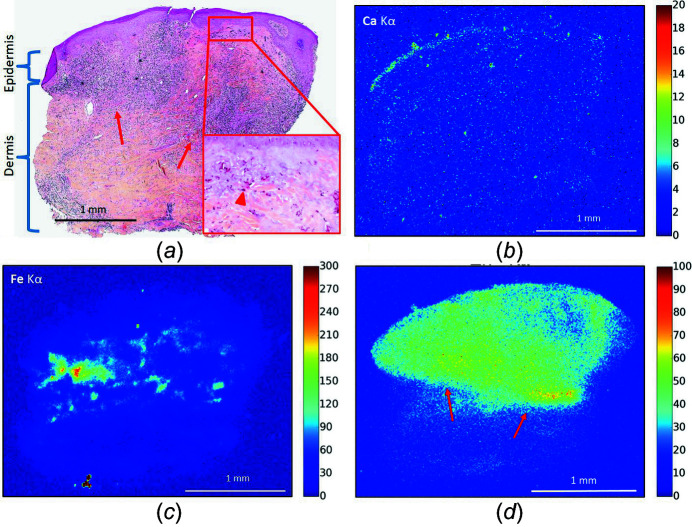
Optical microscopy and XRF maps (patient 2). (*a*) Optical microscopy showing red ink (higher magnification, arrow heads) in the superficial and deep dermis and a diffuse inflammatory infiltrate in the upper dermis (arrows: lower limits of the inflammatory infiltrate), HES-stained (×40 and ×400). (*b*, *c*, *d*) Spatial repartition of Ca, Fe and Zn based on the intensity of the XRF *K*α emission. (*d*) Arrows point to the lower limits of the inflammatory infiltrate, in correlation with panel (*a*). The XRF map was acquired at 10 keV using a 10 µm step size and fast acquisition time (80 ms).

**Figure 3:**
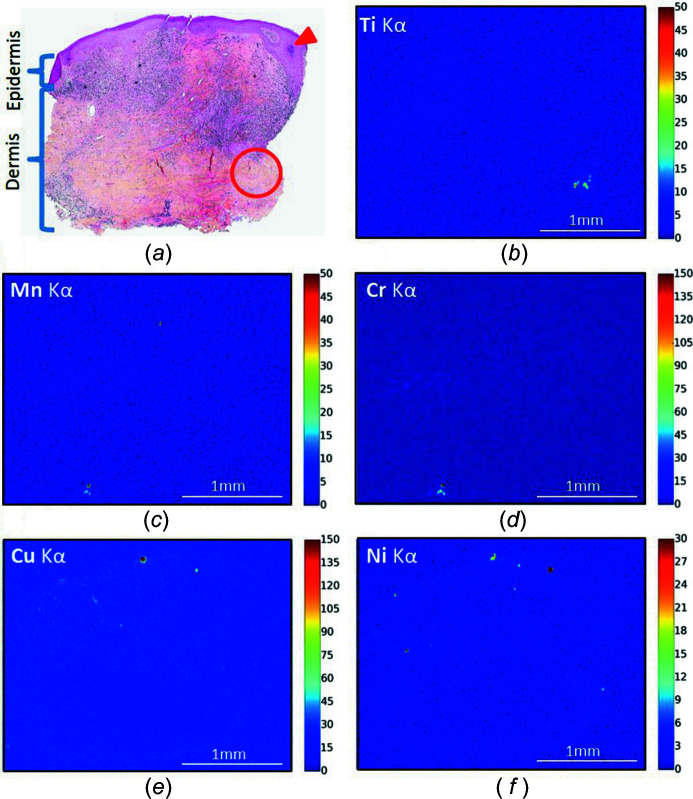
Optical microscopy and XRF maps (patient 2). (*a*) Optical microscopy showing red ink in the superficial and deep dermis and a diffuse inflammatory infiltrate in the upper dermis, HES-stained (×40). Note the upper extremity of a hair follicle (arrowhead), with a circle indicating the area likely to contain the follicular bulb (not visible on this section). (*b*)–(*f*) Spatial distribution of Ti, Mn, Cr, Cu and Ni based on the intensity of the XRF *K*α emission. An XRF map was acquired at 10 keV using a 10 µm step size and rapid acquisition (80 ms).

XRF maps performed at the micrometric scale clearly revealed the distribution of major elements such as Ca, Fe and Zn in most of the samples (Fig. 2 ▸). In addition, various minor elements were identified for the 15 patients: Ti in 11, Ni in 8, Cr in 8, Mn in 7, Cu in 7 and As in 1 (Figs. 3 ▸ and 4 ▸). These metals can have various origins in tattoo inks. For example, recent studies showed that Ti and Cu (among others) are used as colorants and shading, whereas As, Cr and Ni (again among others) tend to be contaminants (Forte *et al.*, 2009*a* ▸,*b* ▸).

These results can be compared with the literature. In an investigation performed on 226 inks using X-ray emission spectroscopy, 15 metals and one halogen were present above the limit of quantification (Tighe *et al.*, 2017 ▸). In order of abundance, these were Ti, Fe, Cr, Cu, Zr, Mn, Br, Ni, Nb, Sr, Zn, Ba, Mo, Pb, V and W (Tighe *et al.*, 2017 ▸). The medical literature indicates quite clearly that several of these elements, namely Ti, Cr, Mn, Ni and Cu, all of which are commonly found in tattoo inks, are associated with allergic reactions (Thyssen *et al.*, 2011 ▸). There is a case of a patient with a local allergic reaction to tattoos associated with systemic symptoms disappearing a week after removal of a metallic implant. XRF analysis showed that both the skin biopsy and the implant contained high levels of Ni, Cr and Ti, suggesting the role of these metals in both the local and the systemic clinical manifestations (Cuyper *et al.*, 2017 ▸).

Some of the metals identified, such as Zn, Fe, Ca, Cu and Cr, are naturally present in human skin, and their detection can therefore reflect endogenous or exogenous origin, while others, such as Ti, Mn, Ni and As, are exclusively exogenous. The micrometre size of the incident monochromatic beam reveals the spatial distribution of these different elements, allowing precise determination of their origin.

Together with correlation to the location of pigment and inflammation observed by optical microscopy, this gives a clue to the exogenous or endogenous origin of Zn, Fe, Ca, Cu and Cr. Indeed, diffuse and homogeneous distribution of these elements probably reflects endogenous origin while a few intense spots co-located with the pigments probably indicates exogenous origin. This can be illustrated by the distribution, from XRF, of the two elements Zn and Ti. Let us discuss the biological significance of these two elements.

**Figure 5:**
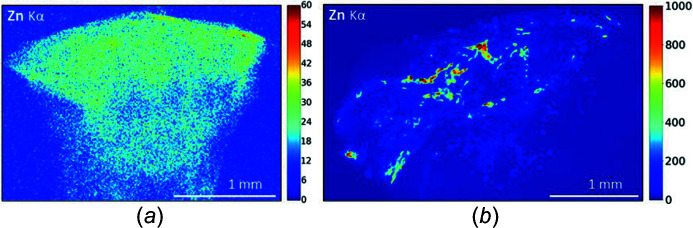
XRF maps, spatial distribution of Zn. (*a*) Patient 11, Zn is diffusely present in the skin tissue, co-located with the inflammatory infiltrate, suggesting an endogenous origin. (*b*) Patient 8, Zn is heterogeneously distributed in the skin tissue, with many intense areas of high Zn concentration, co-localized to the tattoo pigment, suggesting exogenous origin.

Two different Zn distribution patterns are observed. It may be diffusely distributed in the skin tissue and co-located with the inflammatory infiltrate – suggesting an endogenous origin [Fig. 5 ▸(*a*)] – or diffuse within the skin tissue but with a few intense areas of high Zn concentration, co-localized to the tattoo pigment – suggesting a combination of endogenous and exogenous origin within the same sample [Fig. 5 ▸(*b*)].

Zinc is an essential micronutrient (around 2–3 g for a 72 kg body) involved in the regulation of the innate and adaptive immune responses (Gammoh & Rink, 2017 ▸). The spatial correlation of Zn in our samples with the dermal inflammatory infiltrate highlights the role of this element in inflammatory responses.

**Figure 6:**
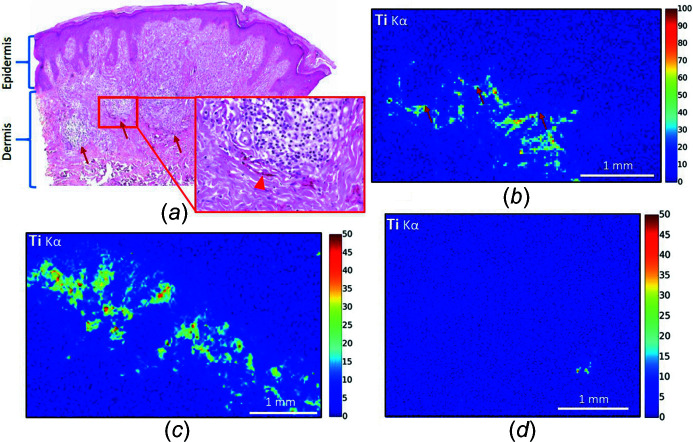
Optical microscopy and XRF maps, spatial distribution of Ti. (*a*) Patient 5, optical microscopy showing red ink (higher magnification HES ×400, arrowheads) in the mid-dermis, at the periphery of a diffused inflammatory infiltrate (HES ×40, arrows: lower limits of the inflammatory infiltrate). (*b*) Patient 5, XRF map, showing Ti on the periphery of the inflammatory infiltrate (arrows: lower limits of the inflammatory infiltrate). (*c*) Patient 6. In these two samples, Ti is co-located with the pigment. (*d*) Patient 2, Ti is located around a hair follicle bulb.

The distribution of Ti, a metal not normally present in the human body, is also interesting. In the samples studied, Ti is usually co-located to the pigment as clearly shown in the XRF maps of patients 5, 6 and 11 [Figs. 6 ▸(*a*), 6 ▸(*b*) and 6 ▸(*c*)], but for one patient (patient 2) Ti was remarkably located in one area of the deeper dermis [Fig. 6 ▸(*d*)]. As a hair follicular orifice was visible directly above this by optical microscopy (Fig. 3 ▸, arrow head), Ti appeared aggregated around a hair follicle bulb in this case.

In this specific case, we can first hypothesize that Ti arises from the tattoo ink and has migrated through the dermis towards the hair follicle after tattooing. Indeed, a migration phenomenon from tattooed skin to lymph nodes has been shown previously for various organic and inorganic tattoo ink substances, including metals such as Ti (Schreiver *et al.*, 2017 ▸; Hayakawa *et al.*, 2018 ▸), Ni (Schreiver *et al.*, 2019 ▸) and Hg (Hayakawa *et al.*, 2018 ▸). Another possibility is that Ti arises from cosmetics applied to the tattoo by the patient, possibly sunscreen, often used to provide sun protection to the tattoo following the procedure and known to contain nanosized particles of Ti (Newman *et al.*, 2009 ▸). Indeed, Ti penetration through the hair follicular orifice has been demonstrated (Lademann *et al.*, 1999 ▸), which might therefore cause Ti accumulation around the hair follicle in patient 2. Furthermore, Ti is suspected of being associated with frontal fibrosing alopecia, an inflammatory skin disease (Kidambi *et al.*, 2017 ▸). In this hypothesis, the association of the sensitizer compounds in the tattoo ink, and Ti from topical application on the tattoo, might have both contributed to the hypersensitivity phenomenon, and potentiated each other, showing the complexity and multifactorial processes underlying these reactions.

### 3.3. Chemical information from XANES spectroscopy

Although the distribution of the metal within the skin biopsy gives us clues regarding its origin as endogenous or exogenous, this should be confirmed by analysis of its precise chemical structure. Indeed, some of the chemical phases in which Zn and Fe are engaged – such as zincite – are not naturally present within the human body, proving that their presence must be due to tattoo inks. To confirm this, XANES measurements were performed on four skin biopsies (patients 2, 8, 13 and 15) at the Zn *K*-edge and on six (patients 2, 5, 10, 11, 13 and 14) at the Fe *K*-edge.

**Figure 7:**
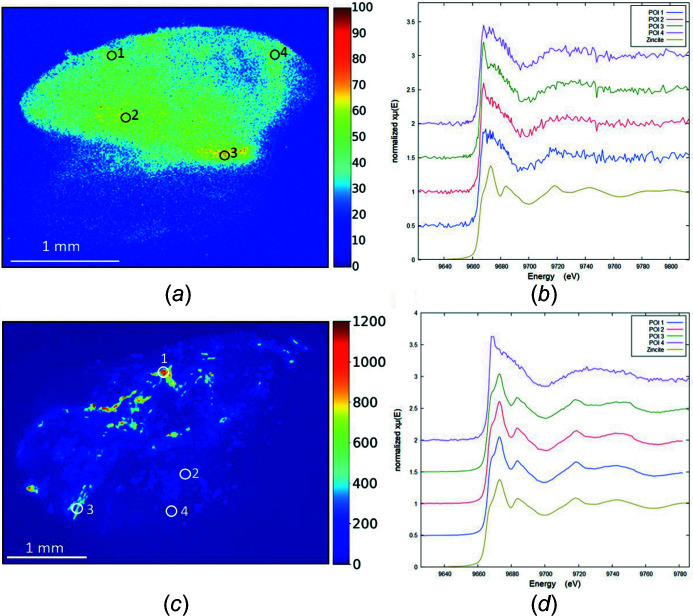
XRF maps with localized POI and XANES spectra at the Zn *K*-edge (patients 2 and 8). A reference spectrum of zincite (ZnO) is also shown as comparison. (*a*, *b*) Patient 2, the four spectra are similar and consistent with endogenous Zn. (*c*, *d*) Patient 8, three spectra are consistent with exogenous Zn (ZnO), and one with endogenous Zn (POI 4).

For example, four XANES spectra were measured at the Zn *K*-edge on points of interests (POI) in the XRF maps of patients 2 and 8 in order to identify the Zn environment (Fig. 7 ▸). Some spectra identify zincite (ZnO), such as three spectra of patient 8. Other spectra (one from patient 8, and those acquired on all other patients) show the maximum of the white line shifted to lower-energy and less structured oscillations, similar in appearance to those observed in human parkin protein (Konovalov *et al.*, 2020 ▸), which contains multiple Zn binding sites, suggesting endogenous Zn associated with dermal proteins.

**Figure 8:**
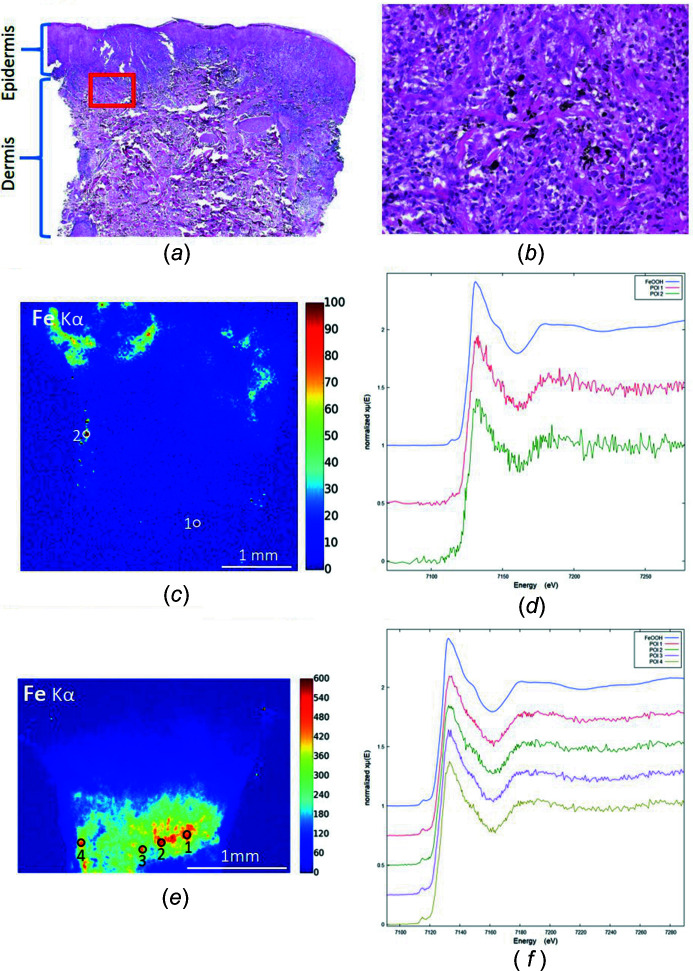
XRF maps with localized POI and XANES spectra at the Fe *K*-edge (patients 10 and 11). A reference spectrum of an iron oxyhydroxide compound (FeOOH) is also shown as comparison. (*a*) Patient 10, optical microscopy HES ×40. (*b*) Patient 10, optical microscopy, HES ×400 [corresponding to the red rectangle on panel (*a*)], with clear identification of dark ink. (*c*) Patient 10, Fe XRF map. (*d*) Patient 10, XANES spectra at the Fe *K*-edge. (*e*) Patient 11, Fe XRF map. (*f*) Patient 11, XANES spectra at the Fe *K*-edge.

As a further example, XANES spectra were acquired at the Fe *K*-edge on POI in the XRF maps of patients 10 and 11 in order to identify the Fe environment (Fig. 8 ▸); all the XANES spectra are similar.

However, a difference is observed as the shoulder at 7150 eV is visible for patients 10 and 7, while it is less visible for patients 11 and 14. In addition, the first oscillation at 7180 eV, related to oxygen neighbors, shows a modified shape. The overall shape of the spectra is similar to that of iron oxy-hydroxides, such as goethite or ferrihydrite compounds, with Fe^3+^ in an octahedral environment. Exogenous Fe oxides, such as magnetite or hematite oxides that have very specific spectral profiles, might be present in these cases (Rueff *et al.*, 2004 ▸; Wilke *et al.*, 2001 ▸).

### 3.4. Chemical information from Raman spectroscopy

**Figure 9:**
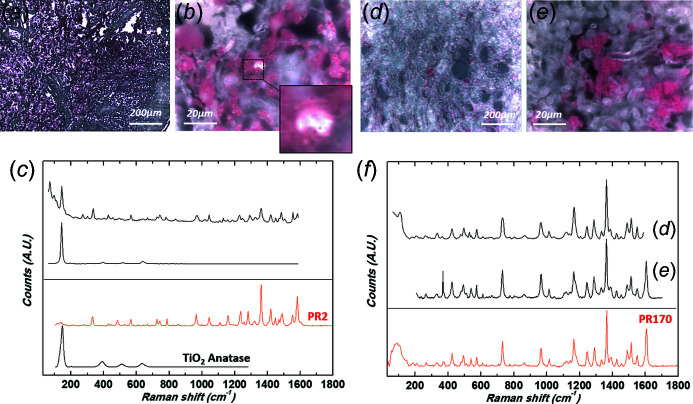
Raman spectroscopy: identification of azo pigments. (*a*, *b*) Optical micrographs of patient 6’s biopsy at two different magnifications [(*a*) 10× and (*b*) 100×] revealing the presence of micrometre-size clusters, mixture of PR2 and TiO_2_ (inset: magnification of a white anatase pigment cluster). (*c*) Corresponding Raman spectra (without correction of the luminescence background). Reference spectra from the Soprano spectral library are also given as comparison (PR2, C.I. 12310, Monoazopigment, Naphthol AS) and anatase (TiO_2_) downloaded from the RRUFF spectral library (ID R110109, 780 nm). (*d*, *e*) Optical micrographs of patients 2 (*d*) and 1 (*e*) revealing the presence of micrometre-size red pigments PR170. (*f*) Corresponding Raman spectra (excitation wavelength λ_exc_ = 785 nm, objective 100×, numerical aperture = 0.9). Both spectra are baseline corrected. Reference spectra from the Soprano spectral library are also given as comparison (PR170 C.I. 12475, Monoazopigment, Naphthol AS).

**Figure 10:**
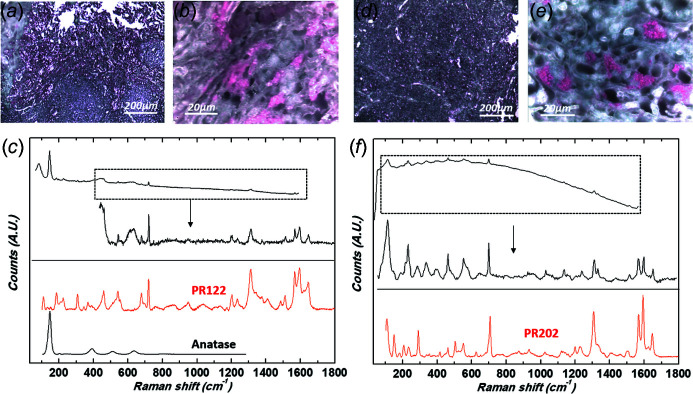
Raman spectroscopy: identification of quinacridone. (*a*, *b*) Optical micrographs of patient 5’s biopsy at two different magnifications [(*a*) 10× and (*b*) 100×] revealing the presence of micrometre-size red and white pigments, mixture of PR122 and TiO_2_. (*c*) Corresponding Raman spectra (top spectrum: no correction of the luminescence background; bottom spectrum: baseline corrected). Reference spectra are also given as comparison: PR122 C.I. 73915, Polycyclic pigment, Quinacridone from the Soprano spectral library, anatase (TiO_2_) downloaded from the RRUFF spectral library (ID R110109, 780 nm). (*d*, *e*) Optical micrographs of patient 8’s biopsy at two different magnifications [(*d*) 10× and (*e*) 100×] revealing the presence of micrometre-size red pigments PR202. (*f*) Corresponding Raman spectra. Top spectrum is presented as collected (no correction of the luminescence background), bottom spectrum is baseline corrected and the spectral range adapted to highlight the signature of the red organic pigment. Reference spectra are also given as comparison: PR202 C.I. 73907, Polycyclic pigment, Quinacridone from the Soprano spectral library.

**Figure 11:**
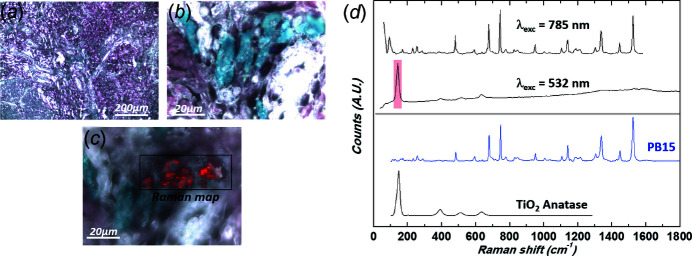
(*a*, *b*) Optical micrographs of patient 4’s biopsy at two different magnifications [(*a*) 10× and (*b*) 100×] revealing the presence of micrometre-size blue pigments PB15. (*c*) Corresponding Raman spectra collected at the excitation wavelength λ_exc_ = 785 nm and 532 nm (top and bottom spectra, respectively, objective 100×, numerical aperture = 0.9). The presence of anatase is revealed using a 532 nm Raman probe as shown in the chemical mapping (red pixels, intensity of the 143 cm^−1^ band) overlapped with the 100× micrograph. Top spectrum is baseline corrected. Reference spectra are also given as comparison: PB15 C.I. 74160, Polycyclic pigment, Phthalocyanine from the Soprano spectral library, anatase (TiO_2_) downloaded from the RRUFF spectral library (ID R110109, 780 nm).

Raman spectroscopy identified six different pigments. Black tattoos were always composed of carbon black, whereas various pigments were identified in colored tattoos. Red tattoos were composed of PR170 (an azo pigment) (where PR is pigment red) in three cases (Scherrer *et al.*, 2009 ▸), PR2 (azo pigment) in two cases (Fig. 9 ▸), PR202 (quinacridone) in one case and PR122 (quinacridone) in one case (Longoni & Bruni, 2021 ▸) (Fig. 10 ▸). The blue tattoo was composed of PB15 (PB is pigment blue) (phthalocyanine) (Fig. 11 ▸).

Black tattoos are the most common, and their main component is carbon black (Jacobsen & Clausen, 2015 ▸). Manufacturing of black inks usually involves thermal combustion of feedstock oil, a process also associated with the formation of PAHs (Regensburger *et al.*, 2010 ▸). Although the carcinogenic potential of PAHs is well known, they are, unlike metals and colored pigments, usually not associated with hypersensitivity reactions to tattoos. In our patients, carbon black was identified in most cases, but, with one exception, it was always associated with a known allergenic metal. The occurrence of skin reactions in this one case (patient 9) is therefore quite unexpected and highlights the complexity of these phenomena. Further studies, analyzing the skin allergenic potential of PAHs, are needed, especially since data suggest that these substances stimulate inflammatory responses and enhance allergic reactions (Nel *et al.*, 1998 ▸).

Azo pigments are the ones most commonly implicated in skin reactions to tattoos (Serup *et al.*, 2020 ▸). Consistent with our findings, quinacridone has also been reported in hypersensitivity reactions (Greve *et al.*, 2003 ▸; Hutton Carlsen *et al.*, 2016 ▸). We also identified a phthalocyanine in one patient, which, to the best of our knowledge, has never been clearly identified in hypersensitivity reaction to tattoo. However, in all the cases except one, these colored pigments were associated with at least one metal with known allergenic properties (either Ti, Cr, Mn, Ni and/or Cu), making the exact identification of the causative compounds impossible without thorough allergenic exploration.

A list of carcinogenic chemicals that should be excluded from tattoo inks was published in a European resolution in 2008 (Council of Europe, 2008 ▸). Although we did not identify any prohibited pigments in our patients, this has not been the case in other studies. A study on 396 tattoos and 55 permanent make-up inks taken from the Swiss market between 2009 and 2017 identified 28 different pigments, four of them prohibited (Niederer *et al.*, 2017 ▸). These authors also compared the pigments found with those declared on tattoo ink labels and convincingly showed that banned pigments are rarely declared, but rather masked by listing non-present legal pigments and label forging. These results highlight the importance of performing rigorous chemical analysis, like ours, of skin reactions to tattoos.

Ti was the metal the most often associated with colored pigments (Figs. 9 ▸, 10 ▸ and 11 ▸), as it was found in all cases except one. Ti is an efficient photocatalyst and can produce reactive oxygen species under UVA irradiation (Chen & Mao, 2007 ▸; Ancona *et al.*, 2018 ▸; Wydra *et al.*, 2015 ▸). In our patients, this metal might have potentiated photodegradation of these colored pigments and promoted metabolic changes, possibly contributing to the formation of sensitizing substances.

Because the photocatalytic properties of these metal oxide deposits depend on their size and form, it is important to provide precise chemical information, as we did using Raman spectroscopy. This allowed us to identify anatase (with a characteristic strong band at 145 cm^−1^ and weaker bands at 395, 517 and 641 cm^−1^) in four cases (Frank *et al.*, 2012 ▸; Wypych *et al.*, 2014 ▸). Recent investigations emphasize that anatase and amorphous forms of nano-TiO_2_ show greater cytotoxicity than the rutile form (Xue *et al.*, 2010 ▸; Huang *et al.*, 2017 ▸).

In a previous study, we identified the association of PR170 with metal oxides (either Ti, Fe or Zn) in three cases of keratoacanthoma, a type of skin tumor (Colboc *et al.*, 2020 ▸). The fact that, in the present study, the same pigment with metal oxide was associated with hypersensitivity but not carcinogenic processes highlights the pathogenic complexity of skin reactions to tattoos.

Interestingly, Raman spectroscopy failed to identify Ti in seven other cases in which it had been identified by XRF. This may be due to strong (auto)fluorescence background, as mentioned earlier. Another explanation may be that, because Raman spectroscopy is performed on a 1.5 µm-thick section of the paraffin-embedded biopsy (and not on the whole biopsy, in contrast to XRF), it failed to identify some of the chemical substances that were indeed present. This highlights once again the importance of using various complementary techniques for precise chemical characterization of tattoo inks, XRF being highly sensitive for elemental detection, while Raman provides information on the nature (composition, structure) of the inorganic (metal oxide) and of the organic compounds.

## 4. Limitations of our study

Because we wanted our study to have a consistent approach from a clinical point of view, we focused on hypersensitivity reactions after the tattoo, thus excluding several cases of infection and skin cancer encountered during this period in the collaborating dermatological centers. This focus necessarily limited the number of samples.

The possibility of chemical modifications of the inks after sample fixation with formol and embedding in paraffin must also be considered. Unfortunately all routine human skin biopsies undergo this process, so such modifications cannot be excluded. However, it is worth pointing out that the color of the formol containing the sample is unchanged after fixation, excluding at least massive dilution of the tattoo inks. Besides, the pigments identified by our techniques faithfully correspond to the color of the tattoo, evidence that their chemistry is unaltered by fixation.

The samples themselves present some challenges. These include: poor regulation of tattoo ink composition, undocumented use of ink mixtures from different manufacturers, and possible photodegradation perhaps accelerated by metal oxide catalysis; these must be acknowledged as significant difficulties in such research.

## 5. Conclusion

We report Raman spectroscopic, XRF and XANES results from skin reactions to tattoos from, to the best of our knowledge, the largest patient cohort yet analyzed. Using these innovative and complementary techniques, we were able to provide wide-ranging information on the organic and inorganic compounds found in skin reactions to tattoos. In each of our patients, we identified various suspected allergens, highlighting the necessity of partnering such studies with allergenic investigations.

XRF identified the metals Fe, Zn, Ti, of Ni, Cr, Mn, Cu and As, and Raman spectroscopy the organic pigments carbon black, PR170, PR2, PR122, PR202 and PB15. Raman spectroscopy also identified the TiO_2_ polymorph, anatase. XANES spectroscopy distinguished exogenous Zn and Fe from these elements of endogenous origin.

Our study also highlights the complexity of tattoo-related reactions and physiological responses: these involve multiple different colored pigments, a variety of organic and inorganic compounds, and diverse histopathological and clinical presentations. Pathogenesis is likely to be multifactorial, including needle trauma during the tattoo procedure, inflammatory reactions, UV radiation, and individual and lifestyle background.

In contrast to previous studies, almost half our patients had reactions to black tattoos, possibly because carbon black was usually associated with allergenic metals such as Ti, Cr and Ni. These results show that modern black inks should therefore be considered as provocative of hypersensitivity reactions as colored inks, as they are unlikely to be pure carbon black.

Additional analyses of skin reactions to tattoos using the same techniques are needed to allow a more reliable definition of the allergenic factors and so help render tattoo ink composition safer.

## Figures and Tables

**Figure 4:**
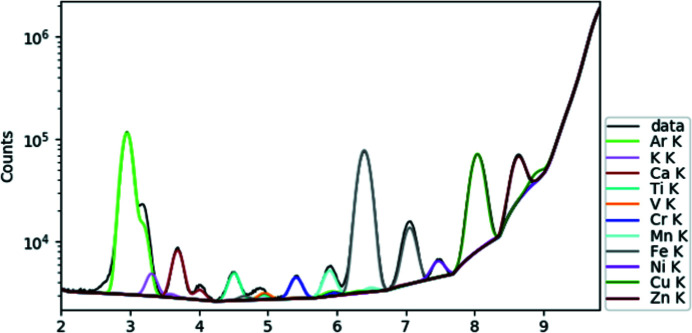
Typical XRF spectrum (patient 2) collected from a biological sample showing the contributions from Ca (*K*α at 3.691 keV, *K*β at 4.012 keV), Fe (*K*α at 6.404 keV, *K*β at 7.058 keV) and Zn (*K*α at 8.638 keV, *K*β at 9.572 keV).

**Table 1:** Patient characteristics and ink composition (N.A., not available; PR, pigment red; PB, pigment blue)

Patient	Sex, age	Color	Skin eruption	Tattoo-to-eruption interval (months)	Histological reaction	Metals	TiO_2_ polymorph	Pigments
1	M, 52	Red	Papular	3	Lichenoid	Fe, Zn, Ti, Mn, Cr	–	PR170 (azo pigment)
2	M, 39	Red	Papular	6	Lichenoid	Fe, Zn, Ti, Mn, Cr, Cu, Ni	–	PR170 (azo pigment)
3	M, 34	Red	Papular	1	Granulomatous	Fe, Zn	–	PR170 (azo pigment)
4	M, 35	Blue	Papular	N.A.	Lympho-histiocytic	Fe, Zn, Ti, Ni	Anatase	PB15 (phthalocyanine)
5	M, 35	Red	Papular	N.A.	Lympho-histiocytic	Fe, Zn, Ti, Cu	Anatase	PR122 (quinacridone)
6	F, 20	Red	Papular	N.A.	Lympho-histiocytic	Fe, Zn, Ti, Cu, Mn, Cr, Ni, As	Anatase	PR2 (azo pigment)
7	F, 21	Red	Papular	N.A.	Lympho-histiocytic	Fe, Zn, Ti	Anatase	PR2 (azo pigment)
8	F, 53	Red	Papular	0.5	Lympho-histiocytic	Fe, Zn, Ti, Mn, Cr, Cu, Ni	–	PR202 (quinacridone)
9	M, 28	Black	Lichenoid	6	Lichenoid	Fe, Zn	–	Carbon black
10	M, 32	Black	Papular	3	Granulomatous	Fe, Zn, Mn, Cr	–	Carbon black
11	F, 21	Black	Papular	Immediate	Lichenoid	Fe, Zn, Ti, Ni	–	Carbon black
12	F, 62	Black	Papular	0.5	Lichenoid	Fe, Zn, Ti, Cr, Cu	–	Carbon black
13	M, 57	Black	Papular	60	Granulomatous	Fe, Zn, Ti, Mn, Cr, Cu, Ni	–	Carbon black
14	F, 30	Black	Papular	14	Granulomatous	Fe, Zn, Ni	–	Carbon black
15	M, 20	Black	Papular	0.5	Lichenoid	Fe, Zn, Ti, Mn, Cr, Cu, Ni	–	Carbon black
